# Diffuse Involvement of Aorta in Patient with Familial Hyperlipidemia

**DOI:** 10.5402/2011/804767

**Published:** 2011-04-12

**Authors:** Mehmet Mustafa Can, Ibrahim Halil Tanboga, Taylan Akgun

**Affiliations:** ^1^GATA Haydarpasa Research and Training Hospital, 81327 Istanbul, Turkey; ^2^Erzurum Education and Research Hospital, 25700 Erzurum, Turkey; ^3^Izmit Seka State Hospital, Izmit 41040, Turkey

## Abstract

Familial hyperlipidemia (FH) is an inherited metabolic disorder caused by low-density lipoprotein (LDL) receptor abnormality. The delayed clearance of serum LDL results in severe hypercholesterolemia, which leads to the accumulation of LDL-derived cholesterol in skin, tendons, and arterial walls.In homozygous form of the disease, severely atheromatous involvement of the aorta extending to the coronary ostia is almost always present, and particular surgical strategy is required to prevent atheroembolic events.

## 1. Case Report

**Figure 1:**
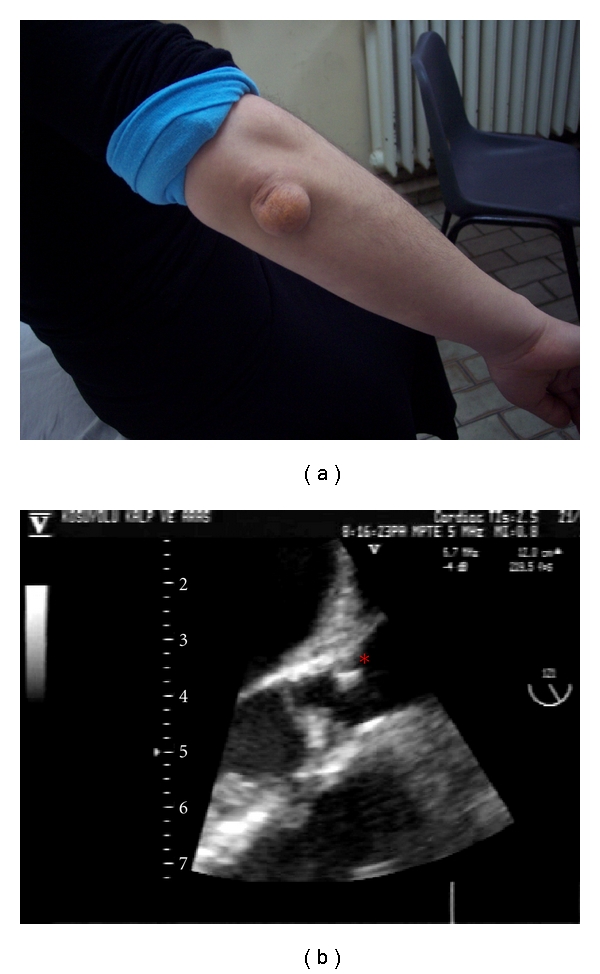
Xanthoma on elbow skin in, (a) an atheroma protruding into the aortic lumen in (b) (red star).

**Figure 2:**
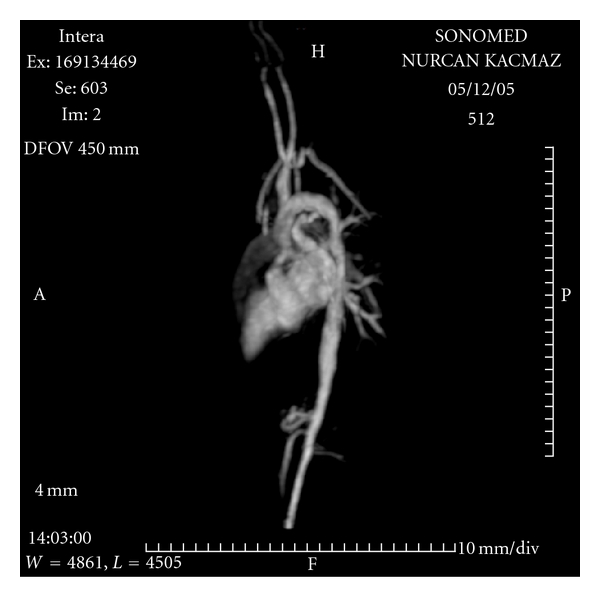
MR angiogram showing diffuse narrowing of the entire aorta.

**Figure 3:**
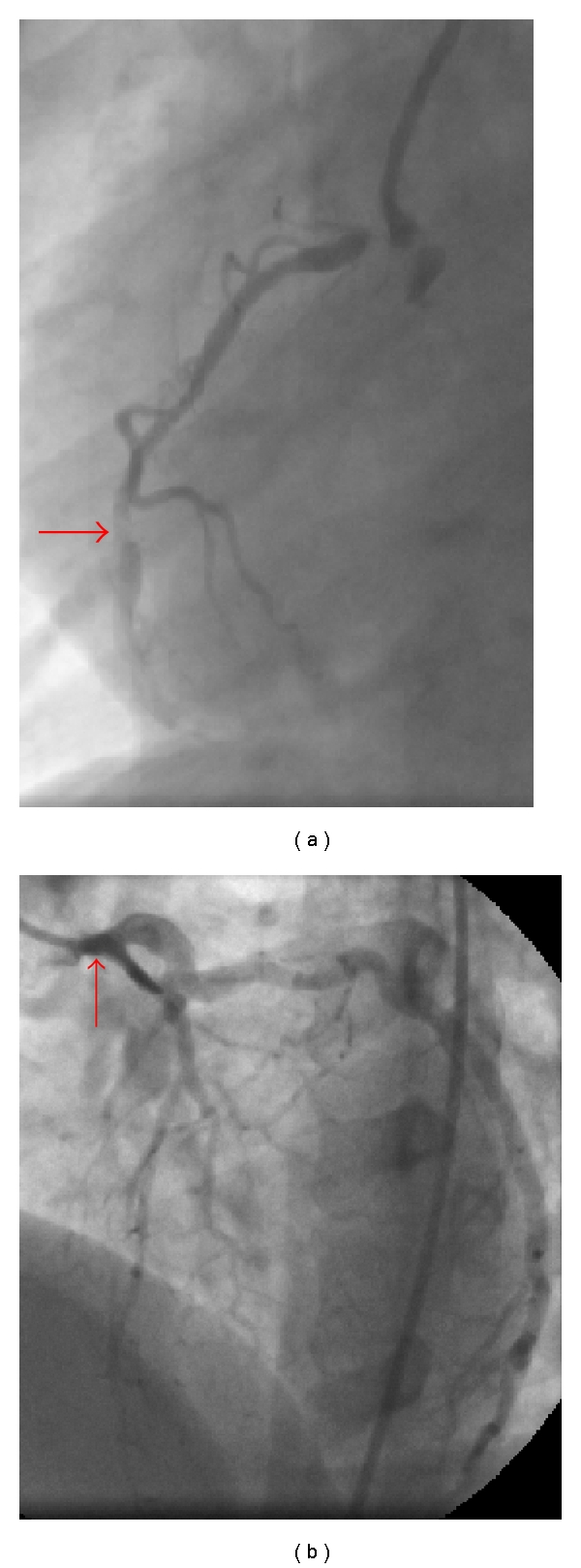
Critical ostial and mid portion stenosis of the right coronary artery and a moderate ostial stenosis of the LMCA on coronary angiography.

A 22-year-old female with homozygous familial hyperlipidemia (FH) presented with symptoms of exertional dyspnea and chest pain. On inspection, the xanthomas on her elbow and knee were rather evident (Figure 1(a)). Precordial auscultation revealed a 3/6 holosystolic murmur over the aortic area, radiating through the neck. The electrocardiogram demonstrated negative T waves in the inferior leads with signs of left ventricular hypertrophy. The levels of low-density lipoprotein (LDL) and high-density lipoprotein (HDL) cholesterol were measured as 450 mg/dL and 48 mg/dL, respectively. Although the aortic valve was structurally normal, aortic transvalvular maximal gradient was measured as 130 mm Hg on transthoracic echocardiogram (TTE). There was also an associating mild aortic regurgitation. The left ventricular outflow tract and proximal ascending aorta could not be optimally evaluated on TTE, because of poor image quality. Transesophageal echocardiogram (TEE), showed the diffuse atheromatous narrowing of the aorta from annular level to the descending thoracic aorta. On TEE, a calcified atheroma, originating from the aortic wall about 1 cm above the aortic annulus, was protruding through the aortic lumen. TEE also showed a diffuse narrowing of the aorta from annular level to the descending thoracic aorta (Figure 1(b)). The diameters of the aorta were measured as 1.1, 1.4, and 1.5 cm at the levels of the aortic annulus, sinotubular junction, and ascending aorta, respectively, (body surface area was 1.56 m^2^). Magnetic resonance angiography confirmed the diffuse narrowing of the entire aorta (Figure 2). Coronary angiograms showed a critical ostial stenosis of the right coronary artery and a moderate ostial narrowing of the left main coronary artery (LMCA) (Figure 3). The patient was referred to surgery. Aortoplasty with autologous pericardial and dacron patches, endarterectomy of the protruding atheroma, and aorto-right coronary artery saphenous bypass grafting were successfully performed. However, despite the success in the surgical procedure, the predischarge control transthoracic echocardiogram performed on the postoperative 10th day revealed a transaortic maximal gradient of 80 mm Hg.

## 2. Discussion

In FH, aortic root is usually the first site of involvement in the process of accelerated atherosclerosis and results in aortic valvular or supravalvular stenosis. Then, the process extends into the coronary ostia [1]. In patients with homozygous FH, supravalvular aortic stenosis (AS) occurs in as many as 44% of cases [2–4]. Supravalvular AS is also seen in heterozygotes with a lower incidence (4 percent in one report) [4]. In surgical procedures of these patients, aggressive aortic palpation, cross-clamping, cannulation, and “sandblasting” effect caused by the jet of blood perfusate from the aortic cannula may cause embolization of atherosclerotic material from the ascending aorta. In a retrospective study, Stern et al. [5] showed that aortic endarterectomy in addition to the coronary artery bypass surgery, resulted in a 3 times higher intraoperative stroke rate. However, in a prospective study, Tunick et al. [6] evaluated the risk of vascular events in patients with protruding aortic atherom**a**s. In this study, 521 patients had TEE (42 had protruding aortic atheromas), and they were followed for up to 2 years. 33% of patients with aortic atheromas had vascular events during followup versus 7% of control group.

In our case, both the diffuse atherosclerotic narrowing of the entire aorta extending into the coronary ostia and the complex (>5 mm in diameter) protruding atheroma were contributing to the symptoms of angina and dyspnea. The transaortic maximal gradient was 130 mm Hg on continuous wave Doppler examination. Aortoplasty with autologous pericardial and dacron patches, endarterectomy of the protruding atheroma, and aorto-right coronary artery saphenous grafting with proximal anastomosis to the relatively nonatheromatous region of the aorta were performed. Postoperative neurological course was uneventful. To avoid an iatrogenic aortic valve insufficiency, the diameters of the reconstructed aorta were kept limited. On postoperative TTE, the transaortic gradient was found to be 80 mm Hg, and pressure recovery phenomenon was speculated to be responsible for this decreased, but still high gradient. Statins and cholesterol absorption inhibitors have only modest effects in reducing cholesterol levels in FH [1]. In this case, the LDL cholesterol level was 450 mg/dL despite high-dose atorvastatin and ezetimibe. Plasmapheresis and liver transplantation could be effective therapeutic options in decreasing LDL cholesterol levels. The patient was discharged under the treatment of atorvastatin 80 mg and ezetimibe 10 mg, and during the follow-up period of 3 years, there were no symptoms or signs consistent with coronary ischemia.
